# Practical guidance to identify and troubleshoot suboptimal DSC-MRI results

**DOI:** 10.3389/fradi.2024.1307586

**Published:** 2024-02-20

**Authors:** Melissa A. Prah, Kathleen M. Schmainda

**Affiliations:** ^1^ Department of Biophysics, Medical College of Wisconsin, Milwaukee, WI, United States; ^2^ Department of Radiology, Medical College of Wisconsin, Milwaukee, WI, United States

**Keywords:** DSC-MRI/Dynamic susceptibility contrast, rCBV/rCBF, perfusion, guide, postprocessing, issues/troubleshooting, brain tumor, CNR/contrast-to-noise ratio

## Abstract

Relative cerebral blood volume (rCBV) derived from dynamic susceptibility contrast (DSC) perfusion MR imaging (pMRI) has been shown to be a robust marker of neuroradiological tumor burden. Recent consensus recommendations in pMRI acquisition strategies have provided a pathway for pMRI inclusion in diverse patient care centers, regardless of size or experience. However, even with proper implementation and execution of the DSC-MRI protocol, issues will arise that many centers may not easily recognize or be aware of. Furthermore, missed pMRI issues are not always apparent in the resulting rCBV images, potentiating inaccurate or missed radiological diagnoses. Therefore, we gathered from our database of DSC-MRI datasets, true-to-life examples showcasing the breakdowns in acquisition, postprocessing, and interpretation, along with appropriate mitigation strategies when possible. The pMRI issues addressed include those related to image acquisition and postprocessing with a focus on contrast agent administration, timing, and rate, signal-to-noise quality, and susceptibility artifact. The goal of this work is to provide guidance to minimize and recognize pMRI issues to ensure that only quality data is interpreted.

## Introduction

The ability to better understand neurodegenerative processes is bolstered by the inclusion of perfusion MR imaging (pMRI) (1–4). Three main acquisitions used in pMRI of the brain are dynamic susceptibility contrast enhanced (DSC), dynamic contrast enhanced (DCE), and arterial spin labeling (ASL) (5, 6). Depending on the acquisition and technique used, the most routinely derived perfusion parameters are relative cerebral blood volume (rCBV) or relative cerebral blood flow (rCBF) (5). While each acquisition technique has varying strengths and weaknesses, the focus of this paper will be on understanding issues associated with perfusion post-processing for DSC acquisition, and how to identify, improve or interpret suboptimal DSC data for the generation of rCBV or rCBF maps (5, 7–9). The information presented herein is intended to serve as a practical guidance for improving DSC acquisition quality and post-processing technique for more reliable and accurate interpretation, providing true-to-life examples of specific issues and potential resolutions, including situations when a DSC acquisition is uninterpretable.

DSC-derived pMRI is based on susceptibility-induced changes in T2/T2* that occur as contrast agent (CA) passes through tissue, where acquisition strategies may include a variety of pulse sequences and scanner settings (5–11). Depending on the overall goal, spin echo (SE) pulse sequences have a primary sensitivity to microvasculature, while more commonly used gradient echo (GRE) pulse sequences produce perfusion maps that are sensitive to all vessels, irrespective of diameter. For gradient-echo acquisition current consensus recommendations consider both low flip-angle (FA) acquisitions, which do not require a preload of CA, and intermediate FA acquisitions, which require a preload of CA, acceptable (10–14). To obtain perfusion data with both types of vessel sensitivity spin and gradient echo (SAGE) sequences have also been developed (15–17).

T1 shortening effects can be present when CA is extravasated into the extracellular extravascular space, which can cause an underestimation of rCBV. Fortunately, giving a preload of CA can minimize the unwanted T1-shortening effects providing a signal from which rCBV can be generated (18–21). When required, it is recommended that the preload dose of CA be given approximately 5–6 min prior to initiation of the DSC sequence (21). However, in the presence of a preload, CA extravasation may then appear in the DSC signal as unwanted T2/T2*-shortening effects. Consequently, for all cases, application of a delta R2*-based mathematical model should be applied that can address T1 and T2 leakage effects (12, 18–20). The most commonly used leakage correction method (19) does address both effects. Regardless, it is necessary to apply leakage correction for both low and intermediate FA approaches. Moreover, if acquisition exceeds 120 s, bidirectional leakage correction may be applied to minimize the effects of CA back flux (22).

While DSC acquisition strategies and leakage correction techniques may improve image contrast and accuracy, evaluation of image quality must also be considered prior to meaningful clinical interpretation. The post-bolus DSC signal profile throughout the brain and in arterial tissue should be characterized, in addition to the evaluation of image noise. Both signal to noise (SNR) and contrast-to-noise (CNR) ratios have been used to characterize voxel-wise image quality (23, 24). SNR evaluates the baseline DSC signal noise, while CNR also considers the post-bolus signal profile. While examining combined GRE and SE sequences, Digernes et al. reported that a voxel-wise contrast-to-noise ratio (CNR) of less than four produces highly unreliable results, where regions of low CNR can falsely overestimate rCBV (24).

Even with the correct acquisition protocol in place, issues specific to DSC acquisition and post-processing quality may occur and generally involve the timing and presence of CA administration, rate of CA administration, noise, and susceptibility artifact. The information presented herein is intended to serve as a practical guide for improving and troubleshooting DSC acquisition quality and post-processing technique for more reliable and accurate interpretation, providing true-to-life examples of specific issues and potential resolutions, including situations when a DSC acquisition is uninterpretable.

## Materials and equipment

All included datasets were collected on either 1.5T or 3T GE or Siemens MR imaging systems. Unless otherwise specified, DSC was collected for 120 s using a gradient-recalled-echo echo-planar imaging (GRE-EPI) sequence using the following prescribed protocol: preload and bolus injection of a gadolinium-based CA followed by a saline flush with TE = 30 ms, TR = 1,250 ms, flip angle = 60°, slice thickness = 5 mm, interslice gap = 1.5 mm, matrix = 96–128 × 96–128, FOV = 220–240 × 220–240 mm^2^ (10). Unless stated otherwise, CA was administered with the use of a power injector at a rate between 3 and 5 ml/s, where the bolus was administered approximately 5–6 min following the preload and at approximately 60 s into the DSC acquisition, so that 30–50 time-points could be obtained for calculation of the baseline signal intensity as recommended (10, 23, 25). In addition to the DSC sequence, a T1w reference image was collected proximate to the DSC acquisition, using the exact same slice prescription as the DSC sequence. This reference image is used to improve co-registration with the anatomic images enabling a more accurate delineation of enhancing tumor on the DSC-MR images. T1w pre- and post-CA images were collected with TR = 7.5–850 ms, TE = 2.3–22 ms, and FA = 40–180°. Information related to the RF coils used and shimming was not available at the time of analysis and therefore cannot be provided.

## Methods

All included data was identified from a database of perfusion MR imaging of brain tumors, where written informed consent was obtained following IRB-approval in accordance with HIPAA guidelines. Cases selected for demonstration included those that contained a T1w CA-enhancing (T1w+C) lesion and highlighted a specific issue relating to DSC-MRI acquisition and/or post-processing. The selected cases fit into one of four categories: (1) Timing and presence of CA administration, (2) rate of CA administration, (3) DSC signal noise, and (4) susceptibility artifact. For all cases, post-processing was performed using FDA-cleared IB Neuro™ and IB Delta Suite™ (Imaging Biometrics, Elm Grove, WI) software plug-ins within the OsiriX MD DICOM viewer (http://www.osirix-viewer.com). Briefly, using a mean of the pre-injection baseline signal together with the DSC signal time course the delta R2* concentration time curves were determined on a per-voxel basis. Note, the first five DSC volumes were discarded, before the determination of the mean baseline signal, due to the expected initial signal transient. Next, leakage-corrected and standardized (19, 26) relative cerebral volume (rCBV) maps were automatically generated. White matter-normalized rCBF maps were also generated using singular value deconvolution of ΔR2* with the arterial input function (AIF) (18, 19, 26, 27). Separate binary regions of interest (ROIs) were generated within artery, tumor, normal-appearing white matter (NAWM), and whole brain. Three voxels were automatically selected and visually verified within arteries for the AIF and were further used for graphical evaluation of the arterial DSC signal profile. Manual adjustments to the AIF location were made when warranted. While visualizing the AIF signal profile is helpful, visualization of the whole brain DSC-MRI profile is most important for verifying that an appropriate DSC-MRI profile has been obtained. NAWM regions of interest (ROIs) approximately 10 mm^2^ in size were drawn on one slice adjacent to the frontal horn. A secondary NAWM location was used when the frontal horn was not appropriate due to the absence of normal-appearing brain in that location and was drawn centrally along the anterior-posterior line and adjacent to the lateral ventricle on the slice where the ventricle appeared most linear. T1w images were co-registered to DSC images via the T1w reference scan. T1w+C lesion ROIs were generated from standardized Delta T1 maps (28) when both pre- and post-T1w images were available, or from manually-thresholded standardized post-CA T1w images when only post-CA T1w images were available. Briefly, following application of image standardization to each, pre-CA T1w images were subtracted from post-CA T1w images to generate Delta T1 maps. T1w+C lesion masks were then obtained from thresholded Delta T1 maps, which exclude blood products and proteinaceous materials (28). Whole brain masks were automatically generated within IB Neuro's built-in masking functions. DSC time series, perfusion maps, and ROIs were exported as DICOM files and converted to NIfTI format using FreeSurfer image analysis suite, which is documented and freely available for download online (http://surfer.nmr.mgh.harvard.edu/). Analysis of functional neuroimaging (AFNI) software (29) was then utilized to visualize the DSC signal time-course in respective tissue types. In-house scripts utilizing AFNI functions were used to generate voxel-wise maps of the signal-to-noise ratio (SNR) and temporal SNR (tSNR) of the DSC series and contrast-to-noise ratio (CNR) (24) of the concentration-time curve (Δ*R*2(*t*)) for all datasets. The voxel-wise SNR and tSNR maps were calculated as follows:

(1) $$\mathrm{SNR}=\frac{\mu_{\mathrm{BL}}}{\sigma_{\mathrm{BL}}}$$

(2) $$\mathrm{tSNR}=\frac{\mu_{\mathrm{BL}}-\delta_{\mathrm{BL}}}{\sigma_{\mathrm{BL}}}$$

where *μ*, *σ*, and δ are the mean signal, standard deviation, and minimum pre-bolus baseline DSC signal, respectively. Voxel-wise CNR maps were calculated from the pre-bolus baseline DSC signal (*S*_BL_) and maximum concentration-time curve value (Δ*R*2*(*t*)):

(3) $$S_{\mathrm{BL}}=\frac{1}{N_{\mathrm{BL}}}\sum_{\;j=1}^{N_{\mathrm{BL}}}S_{j}$$

(4) $$\Delta R2^{*}(t)=-\frac{1}{\mathrm{TE}}\ln\left(\frac{S(t)}{S_{\mathrm{BL}}}\right)$$

(5) $$\mathrm{CNR}=\frac{\Delta R2_{\mathrm{Max}}}{\sigma_{\mathrm{BL}}}$$

where *N*_BL_ is the number of baseline timepoints, *S_j_* is the *j*th image in the DSC time-series, TE is the echo time, and *S*(*t*) is the DSC signal time-course (24). In Eq. 5, Δ*R*2_Max_ is the maximum Δ*R*2 signal and *σ*_BL_ is the standard deviation of the ΔR2 baseline signal (24).

## Results

Data from 21 patients [16 male and 5 female; average age = 52 (22–71) years] previously diagnosed with a grade 2–4 glioma (*n* = 18) or metastatic (*n* = 3) brain tumor were identified for inclusion based on the noted image quality of the pMRI data. One patient's imaging data was used for two separate figures, spaced several years apart. Four of the glioma patients were imaged prior to any treatment, while the remaining had all received standard chemoradiation therapy with/without adjuvant temozolomide prior to various second-line therapies including bevacizumab (*n* = 5), carmustine (*n* = 1), isotretinoin (*n* = 1), tumor-treating fields (*n* = 1), reirradiation (*n* = 2), or clinical trial (*n* = 2) prior to MR imaging. The three patients with metastatic disease had received gamma knife with bevacizumab (*n* = 1) or whole brain irradiation with or without systemic therapy (*n* = 2) prior to MR imaging. In total, 25 DSC exams were acquired with 14 using a GE system (*n* = 10 at 1.5T and *n* = 4 at 3T) and 11 using a Siemens system (*n* = 5 at 1.5T and *n* = 6 at 3T). All figures contain an anatomical image, perfusion maps (rCBV, rCBF) overlaid on anatomical images with 98% opacity, DSC signal profiles from artery, T1w+C lesion, NAWM, and whole brain. DSC signal profiles are displayed for individual tissue types on independent scales from 0 to 1, relative to the maximum value. Whole brain and T1w+C lesion metrics (mean with range) within the DSC signal profile are also reported and include SNR, tSNR, CNR, and percentage of voxels with CNR > 4. All standardized rCBV maps are displayed using an intensity scale from 0 to 8 (arbitrary units), and normalized rCBF maps displayed using an intensity scale from 0 to 12 (arbitrary units).

### Properly acquired DSC MRI data

**Figure 1:**
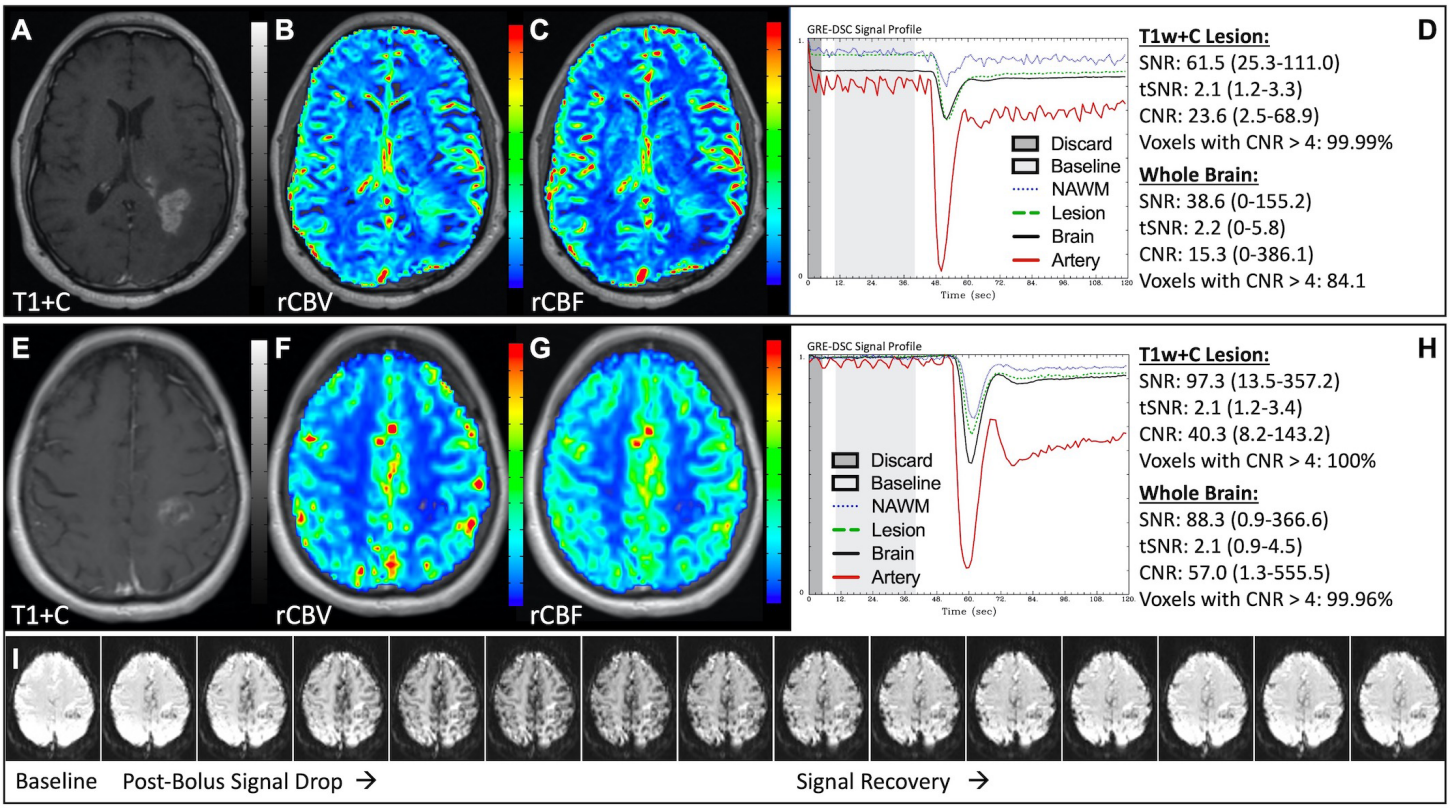
Proper DSC acquisition. Images are from a 48 y/o male with WHO 2021 grade 4 glioblastoma (**A–D**) and a 71 y/o female with history of metastatic small cell lung cancer (**E–I**). Pictured are post-CA T1w (**A,E**), rCBV (**B,F**), and rCBF (**C,G**) images and corresponding DSC signal profiles (**D,H**) in T1w+C lesion, NAWM, artery, and whole brain for two patients with correctly acquired DSC imaging. Also displayed in (**I**) are a subset of DSC images across time from the same patient in (**E–H**) showing the change in signal intensity during and after CA injection. In (**I**), the reduction in signal (darkened blood vessels) occurs in the presence of CA, and is useful for confirmation of CA administration.

**Figure 2:**
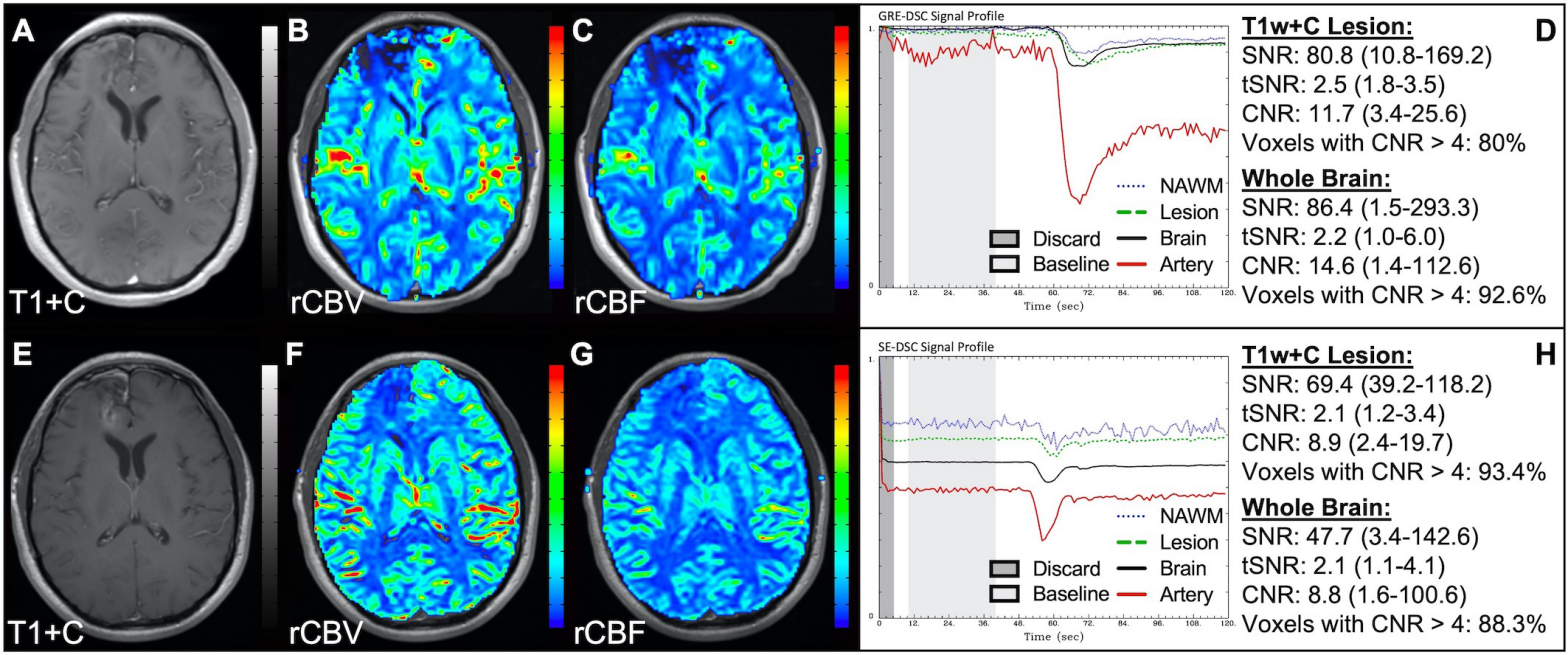
GRE and SE acquisitions. Images are from a 43 y/o male with WHO 2007 grade 3 anaplastic astrocytoma. Pictured are post-CA T1w (**A,E**), rCBV (**B,F**), and rCBF (**C,G**) images and corresponding GRE (**D**; TE = 30 ms) and SE (**H**; TE = 80 ms) DSC signal profiles collected in the same patient within 2 months. GRE DSC (**B–D**) has a much higher SNR than SE and provides a greater sensitivity to larger diameter vessels while SE DSC (**F–H**) is primarily sensitive to microvessels.

**Figure 3:**
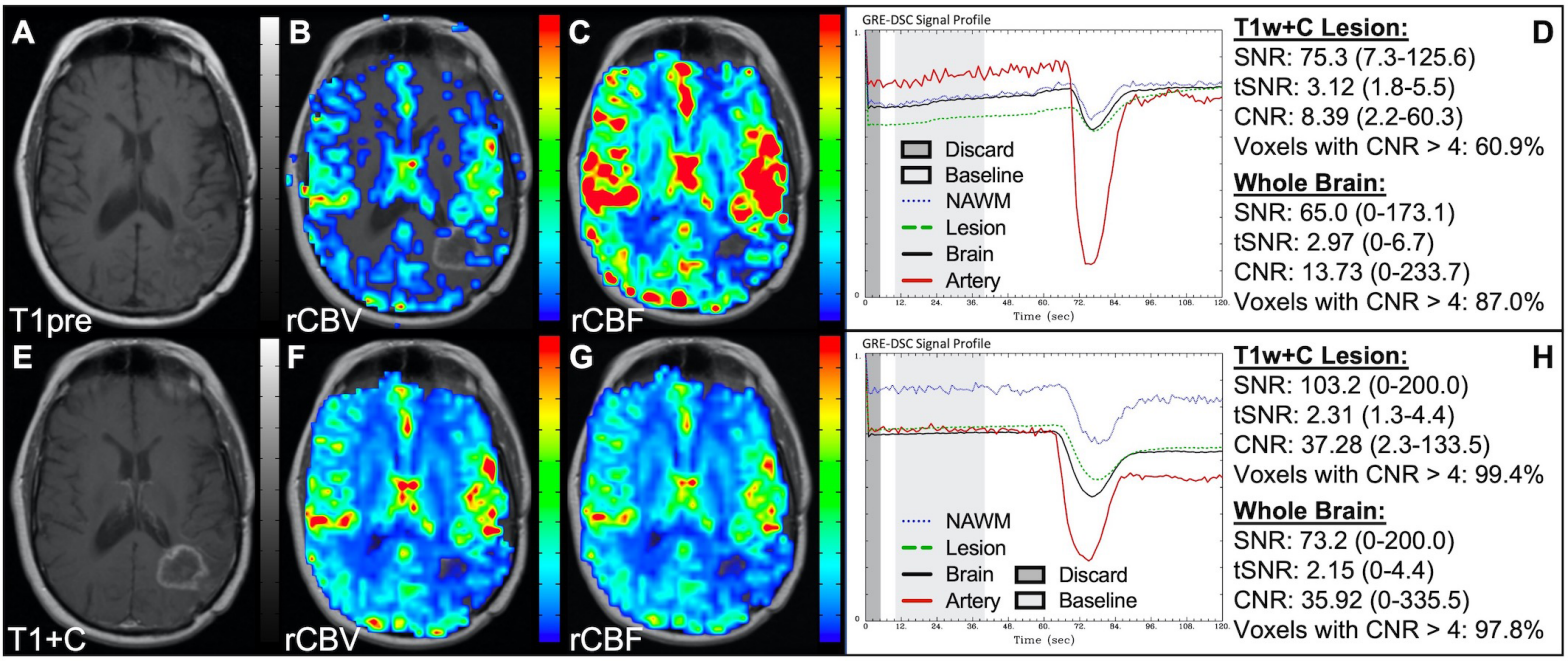
Importance of CA preload. Images are from a 52 y/o male with history of (pre-WHO 2007) grade 2 mixed oligoastrocytoma. Pictured are pre (**A**) and post-CA (**E**) T1w, rCBV (**B,F**), and rCBF (**C,G**) images, and corresponding DSC signal profiles without (**D**) and with (**H**) preload CA administration collected during the same scanning session with identical TE (30 ms) and FA (90°). The rCBV map quality is compromised (**B**) when a large flip angle acquisition is used without the administration of a CA preload.

Figure 1 illustrates properly acquired DSC data and resulting rCBV and rCBF maps in two patients. In both cases, CA was administered following the collection of a sufficient number of baseline timepoints and at a rate allowing for a tight post-bolus signal drop with a sufficient number of post-bolus timepoints to enable the estimation of the leakage effects (19). CNR is greater than 4 in more than 99% of T1w+C lesion voxels and 84% of whole brain voxels. It is important to note the post-bolus signal drop in all tissues, confirming passage of CA rather than noise. Another way to confirm the presence of CA is to verify the DSC signal reduction within blood vessels in the raw data, which exhibit a transient darkening (Figure 1I). A comparison of GRE and SE images acquired in the same patient within 2 months is shown in Figure 2. Both sets of DSC images are acceptable for sufficient calculation of rCBV and rCBF, even though the SE sequence produces a weaker post-bolus signal profile with lower SNR and CNR. Visually, while SE rCBV images are primarily sensitive to microvessels, both methods retain some sensitivity to large diameter vessels, but with GRE showing a greater sensitivity to large diameter vessels. Figure 3 includes two DSC datasets collected in the same patient during the same scanning session with identical TE (30 ms) and FA (90°). While unnecessary for low FA methods (30°) (10), it is recommended that a preload of CA be administered when using intermediate (60°) to high (90°) FA approaches. As shown, lower quality rCBV may result when DSC data is obtained using an intermediate to high flip-angle without a preload dose of CA, despite application of post-processing leakage correction. This result is consistent with previous preclinical (20) and clinical studies (18) using high FA acquisitions. Both preload and post-processing leakage correction were required for greatest accuracy. Furthermore, the global CNR is improved in the acquisition containing a preload dose of CA, where 39% of T1w+C lesion voxels are uninterpretable for the DSC signal acquired without a CA preload.

### Timing and presence of CA administration

**Figure 4:**
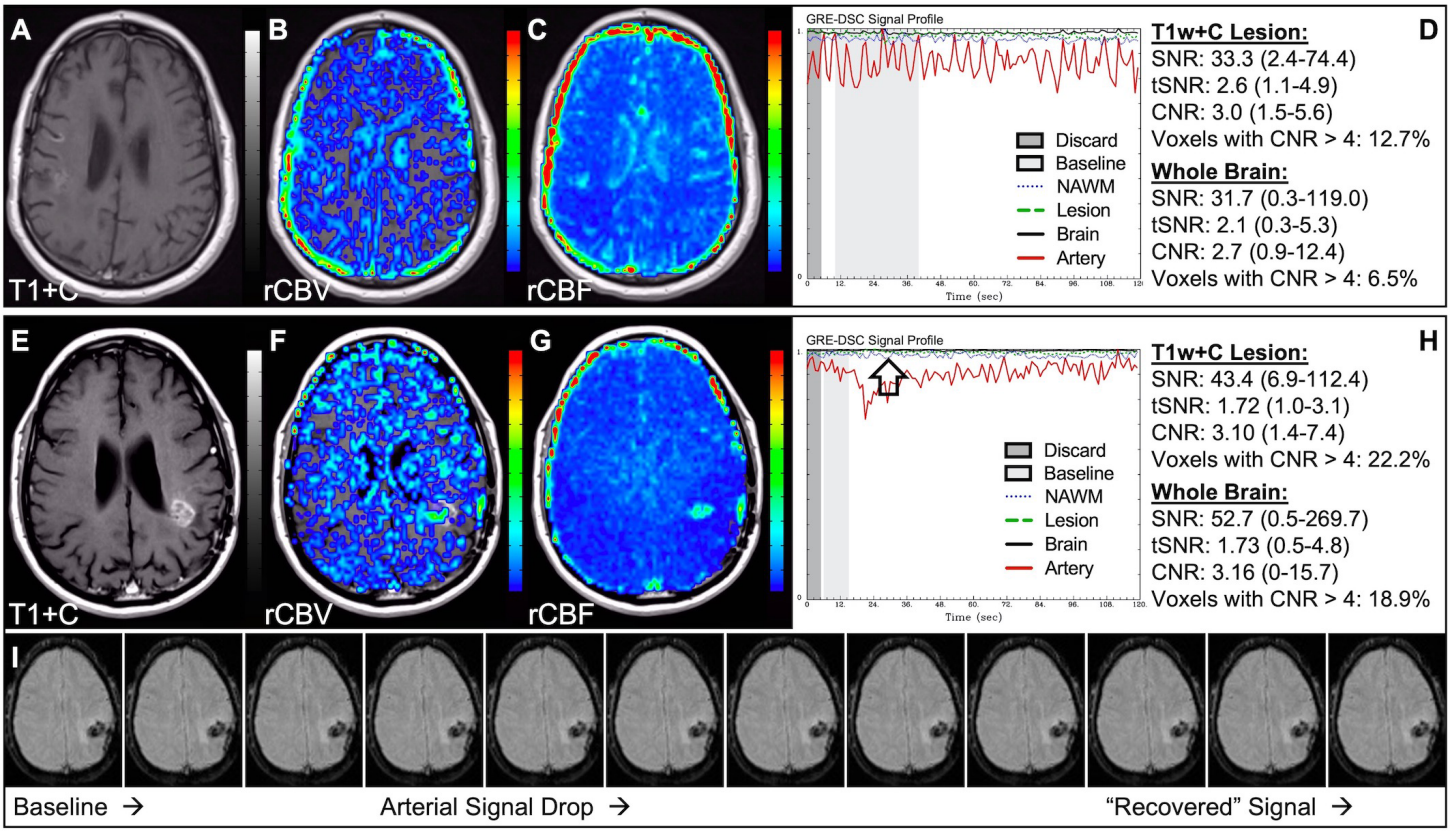
Failed CA administration. Images are from a 55 y/o male with WHO 2007 grade 4 glioblastoma (**A–D**) and a 50 y/o male with WHO 2007 grade 2 oligodendroglioma (**E–I**). Pictured are post-CA T1w (**A,E**), rCBV (**B,F**), and rCBF (**C,G**) images and corresponding DSC signal profiles absent CA administration (**D,H**). It is clear no CA was administered based on the generally flat signal profile in (**D**). While possibly mistaken for a post-bolus signal change in (**H**), the early dip in arterial signal is merely noise, and can be confirmed by comparison to the flat signal in other tissues (black arrow). Additionally, the DSC images (**I**) do not show a signal loss (darkening) in blood vessels during the arterial signal drop. Furthermore, for both patients, an abnormal speckled appearance is seen for rCBV and flat contrast seen for rCBF.

**Figure 5:**
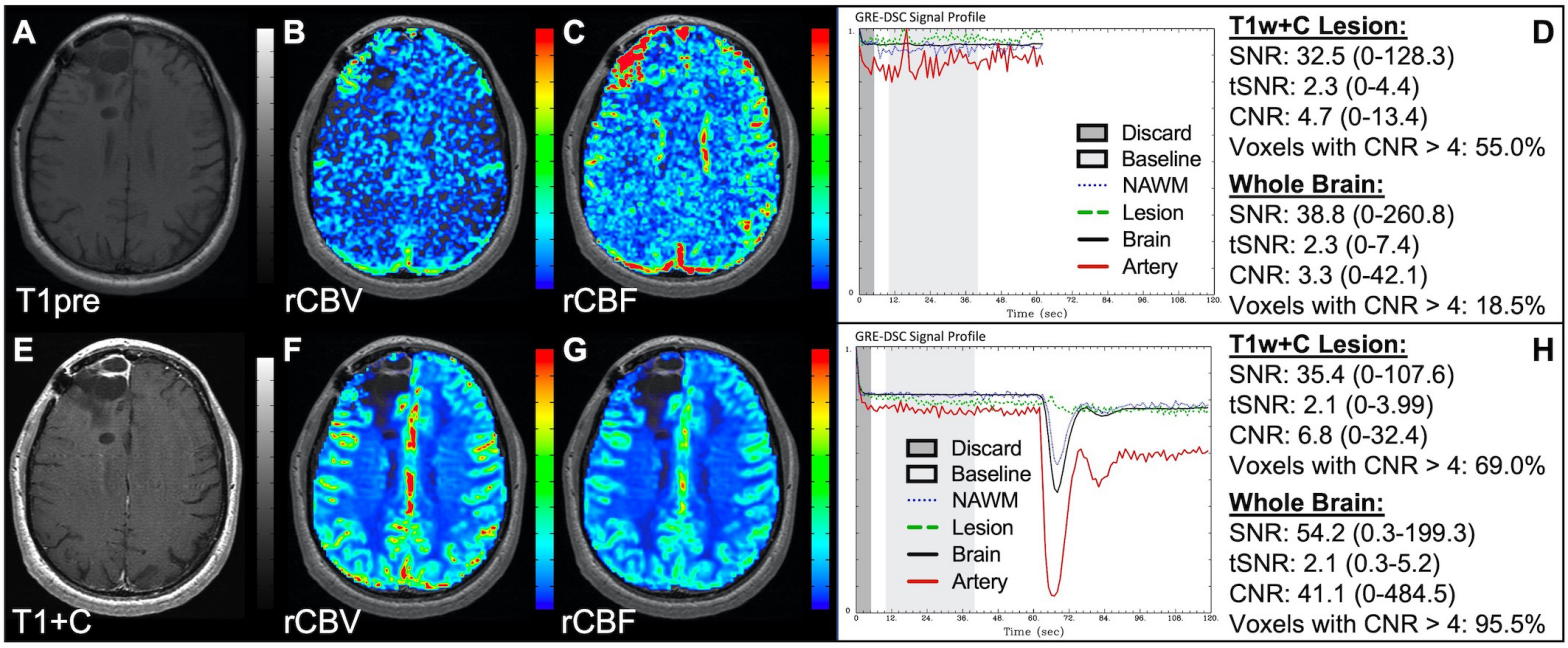
CA timing. Images are from a 53 y/o male with WHO 2007 grade 3 anaplastic astrocytoma. Pictured are pre (**A**) and post (**E**) contrast T1w, rCBV (**B,F**), and rCBF (**C,G**) images, and corresponding DSC signal profiles (**D,H**), respectively. An injection timing error during low FA (30°) DSC acquisition (**B–D**) was mitigated by collecting a mid-range FA (60°) DSC acquisition (**F–H**) using a second dose of CA during the same scanning session.

**Figure 6:**
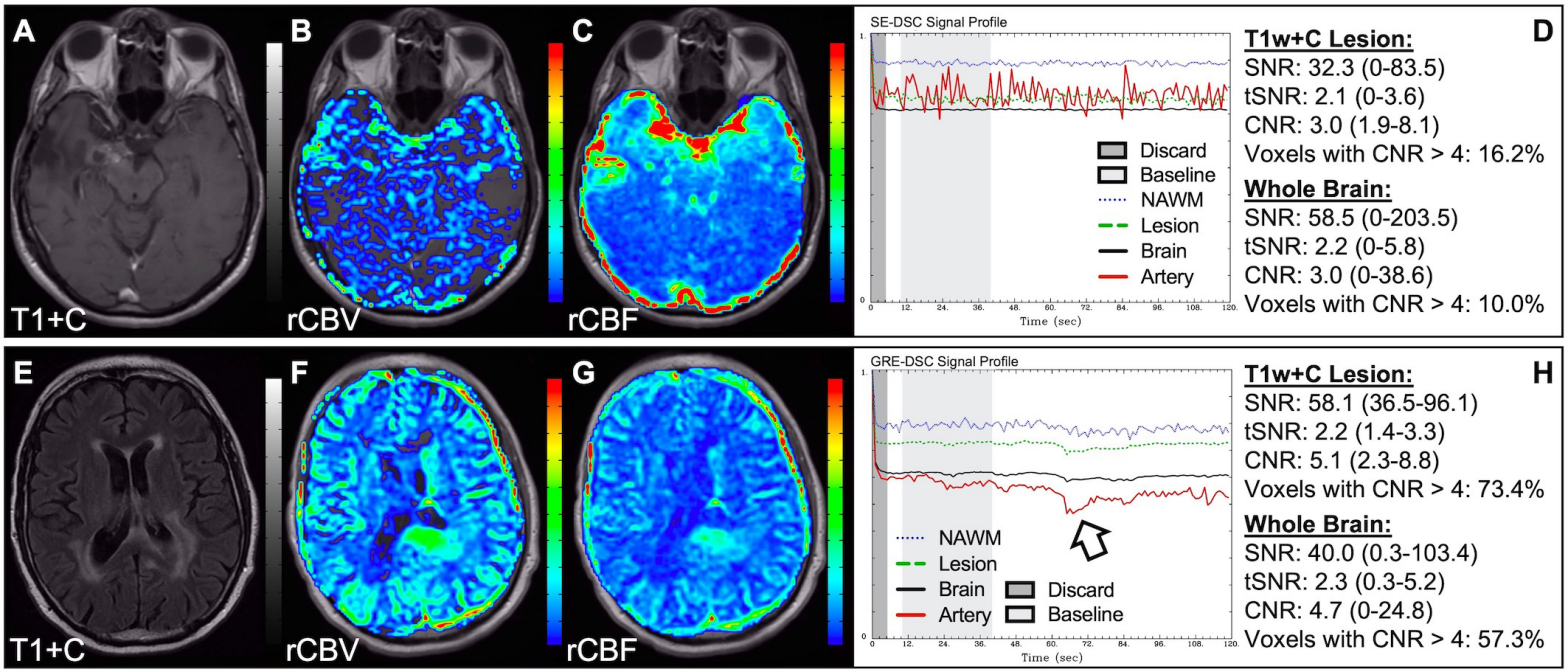
Absent CA changes despite injection. Images are from a 36 y/o male with (pre-WHO 2007) grade 2 astrocytoma (**A–D**) and a 68 y/o male with WHO 2007 grade 2 astrocytoma (**E–H**). Pictured are post-CA T1w (**A**), FLAIR (**E**), rCBV (**B,F**), and rCBF (**C,G**) images and corresponding SE (**B–D**, TE = 80 ms) and GRE (**F–H**, TE = 30 ms) DSC signal profiles, where CA was not properly administered due to an IV leak (**D**) or because the IV line was clamped but allowed some CA to enter (**H**) This can be recognized by the lack of DSC signal drop in brain and correspondingly low CNR across the brain. While the perfusion data in (**F–H**) is not trustworthy, follow-up DSC imaging corroborated the finding of hyperperfusion in the T1w+C lesion owing to the robustness of rCBV calculation despite a DSC signal profile resulting from a weak post-bolus CA injection (arrow).

Figure 4 demonstrates two cases where the CA administration failed during the DSC-MRI acquisition. This can occur when there are problems with the power injector or compromised venous access. Both cases have dreadfully low CNR throughout the brain as 81+% of voxels are uninterpretable. In addition, while the case in the bottom row appears to have an arterial drop in signal, this may be attributed to motion since the DSC signal profile in all the other tissues, for both cases, is flat and noisy confirming a lack of true CA changes. This is further confirmed by examining the DSC images for presence of CA changes. For example, the blood vessels in the DSC images at the time of arterial signal drop do not appear darkened as occurs when CA is present. Rather, the images obtained during the image collection for baseline images, at the time of the sporadic arterial signal change, and during what should be the time of signal recovery show no visual difference. Under such circumstances, the rCBV maps can have a speckled appearance while the rCBF maps show no contrast across the brain. In Figure 5, a low FA DSC sequence was attempted, but with mis-timed CA administration so that CA changes were not captured during the abbreviated acquisition. The images in the top row mirror those in Figure 4 with the characteristic speckled appearance and flat DSC signal profile. The acquisition was stopped but, because CA was already injected, a second attempt at acquiring DSC could proceed (approximately 6 min after initial CA delivery) with an intermediate FA approach resulting in an acceptable perfusion study. Protocols that employ the no preload low FA approaches allow for another opportunity to collect DSC images should an error in the timing of CA administration occur. Similarly, Figure 6 shows two cases where the technician injected CA at the appropriate time, yet relatively flat DSC signal profiles and low CNR throughout the brain and T1w+C lesion resulted. For the case in the top row, a follow-up investigation revealed that the IV leaked resulting in no CA being administered to the patient. Likewise, for the case in the bottom row, the IV line was inadvertently clamped, but allowed some CA to slowly enter and produce a very weak CA change in the DSC signal profile. While the perfusion maps in the first case are uninterpretable, the weak CA change in the signal profile in the second case is severely compromised and should be interpreted with caution.

### Rate of CA administration

**Figure 7:**
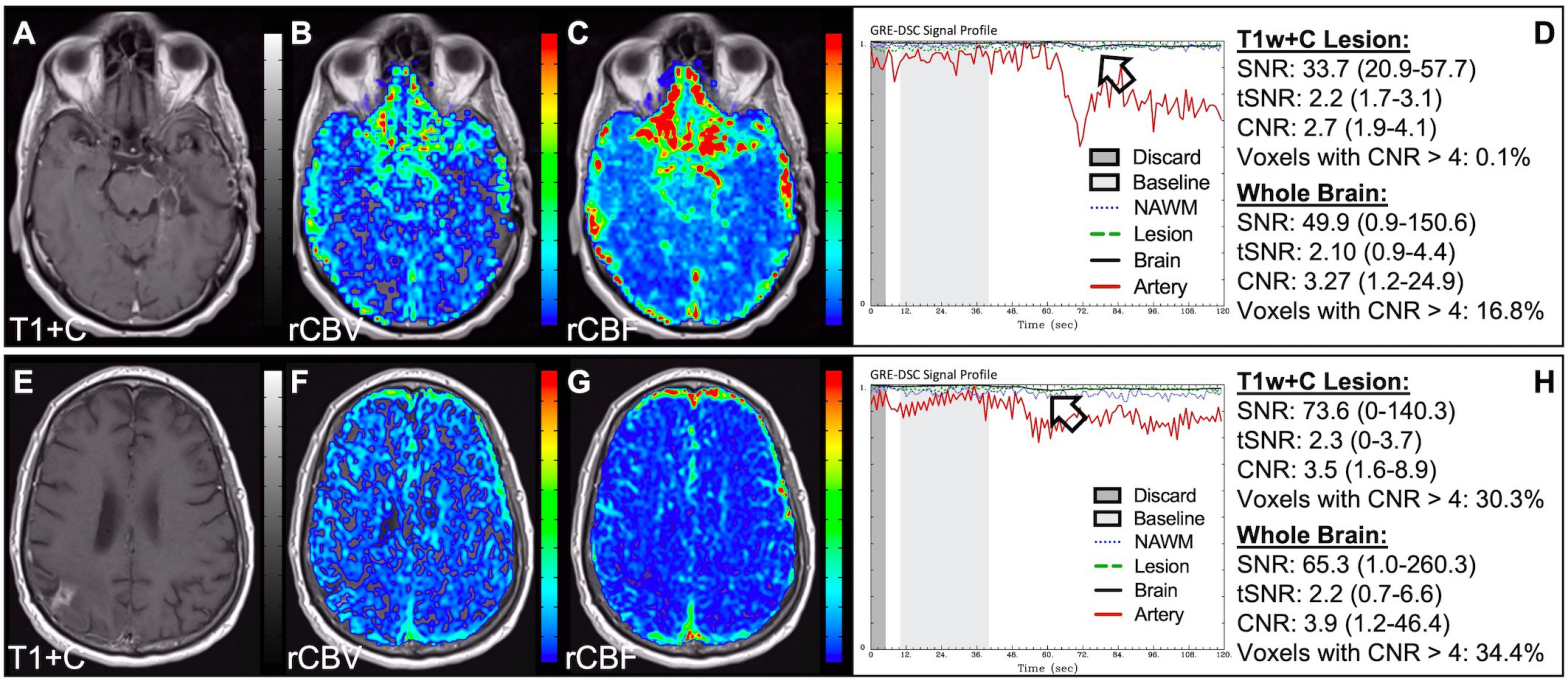
Shallow CA administration. Images are from a 70 y/o male with WHO 2007 grade 4 glioblastoma (**A–D**) and a 67 y/o male with metastatic lung cancer (**E–H**). Pictured are post-CA T1w (**A,E**), rCBV (**B,F**), and rCBF (**C,G**) images and corresponding DSC signal profiles (**D,H**) following a noisy, weak injection of CA (**D**). In both cases the images are uninterpretable as the CA signal drop throughout the whole brain (arrow) is not sufficiently present to allow for calculation of perfusion maps. Note that the rCBV maps have poor CNR and display a speckled appearance as seen in cases where CA wasn't properly injected during acquisition.

**Figure 8:**
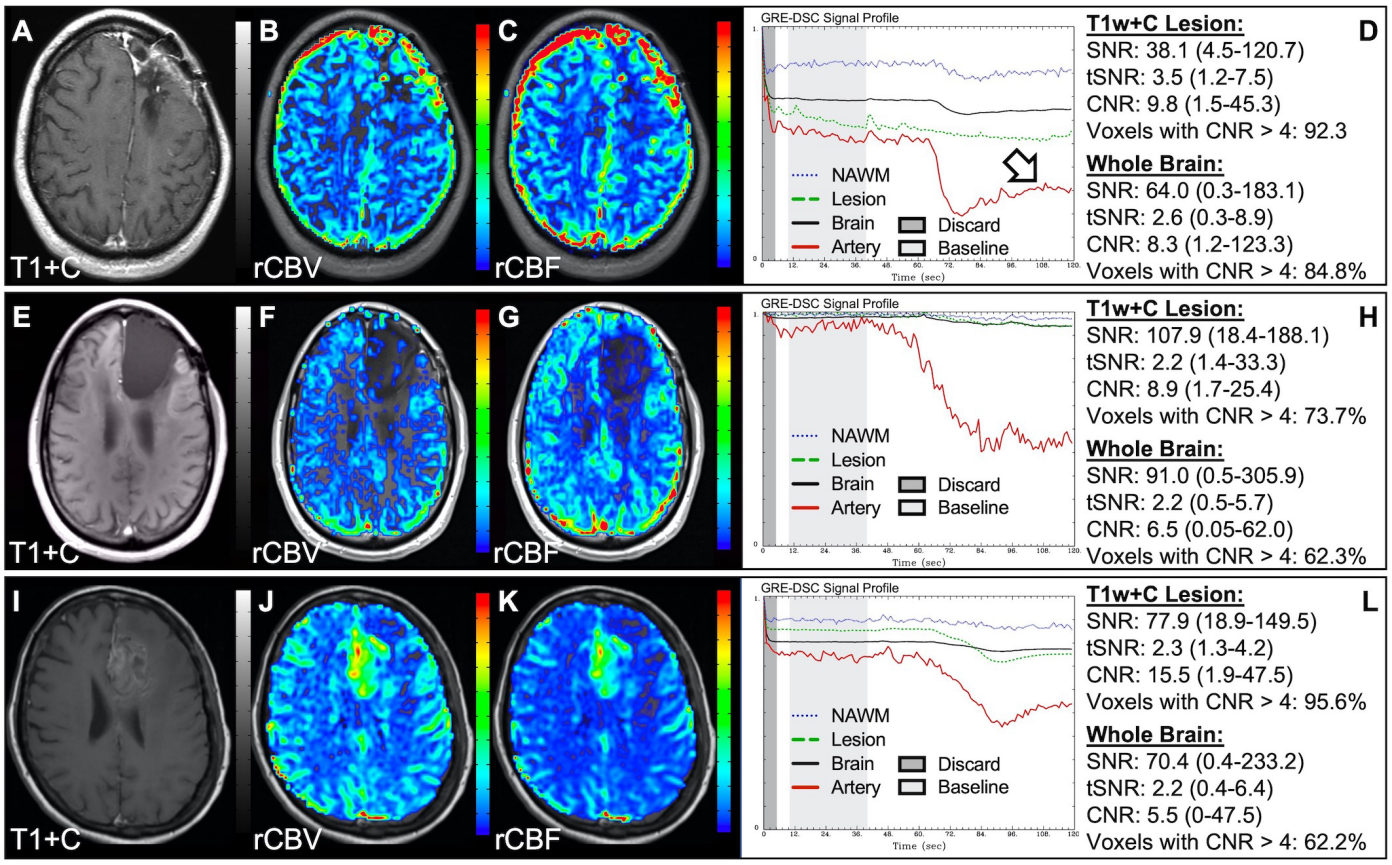
Inconsistent and delayed CA injection. Images are from a 22 y/o male with WHO 2016 grade 2 diffuse astrocytoma (**A–D**), 53 y/o male with WHO 2007 grade 4 glioblastoma (**E–H**), and 63 y/o female with WHO 2007 grade 4 glioblastoma (**I–L**). Pictured are post-CA T1w (**A,E,I**), rCBV (**B,F,J**), and rCBF (**C,G,K**) images and corresponding DSC signal profiles obtained during inconsistent and slow rates of CA administration (**D,H,L**). While suboptimal, the DSC acquisition in (**D**) has substantial recovery (arrow) and is therefore still suitable for rCBV calculation as it is dependent on the area under the curve. This may not be the case for rCBF which is more sensitive to bolus delay and dispersion. DSC acquisitions shown in (**H**) and (**L**) are not acceptable for perfusion calculation as the slow rates of injection also impede the ability of capturing a sufficient return towards baseline signal.

**Figure 9:**
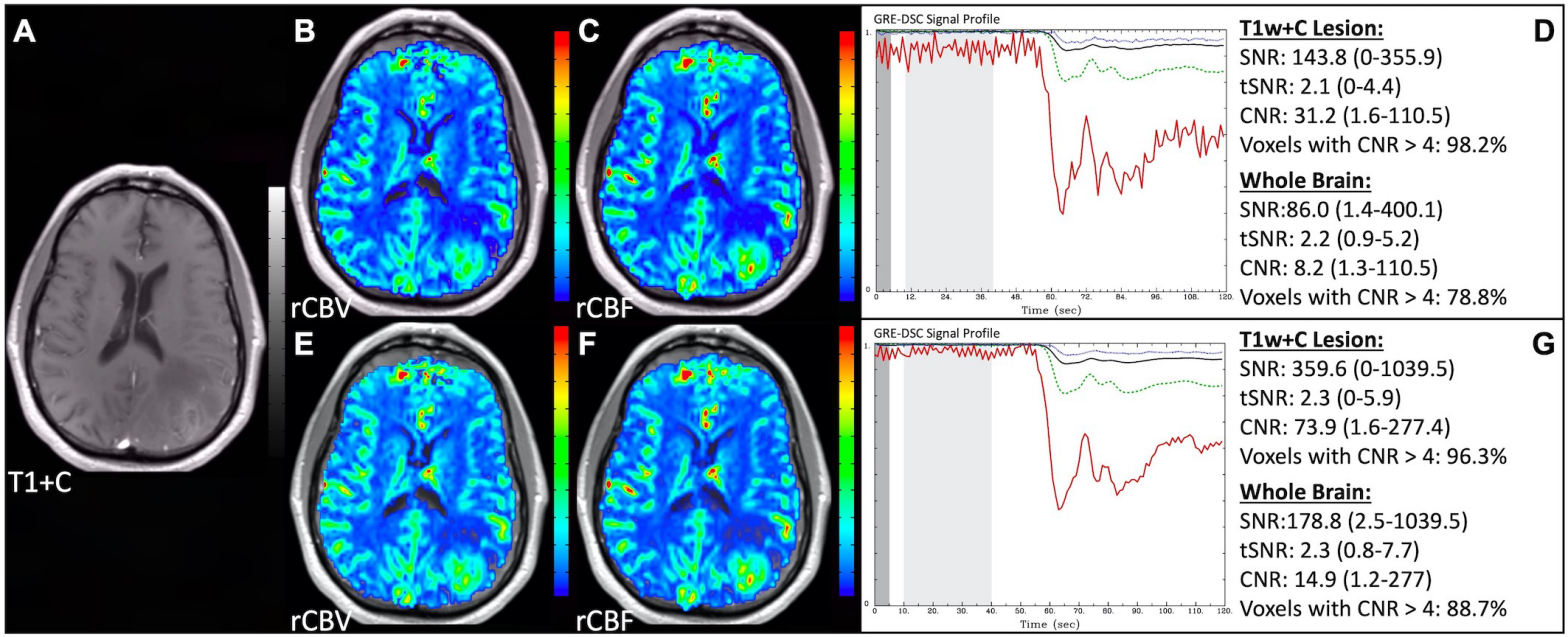
Irregular CA injection. Images are from a 64 y/o male with WHO 2007 grade 4 glioblastoma (**A–G**). Pictured are post-CA T1w (**A**), rCBV (**B,E**), and rCBF (**C,F**) images and corresponding DSC signal profiles (**D,G**) where CA was administered in an irregular pattern prior to (**D**) and following (**G**) motion correction. Irregular appearing injections have sometimes been observed with manual CA injection or when the power injector sticks. Despite being suboptimal, adequate rCBV might still be determined since the calculation depends on the area under the curve. However caution should employed as the signal irregularity might be caused due to motion, which should not be integrated as it is not representative of true signal changes. In this case, motion correction did not considerably alter the resulting perfusion maps further confirming these were true signal changes.

In situations where DSC signal is poor, yet CA was administered and timed correctly as indicated by an acceptable arterial DSC profile, with no obvious issues with the IV or power injector, the patient may have poor vasculature such as can occur with, for example, chronic drug abuse, Ehlers-Danlos syndrome, or peripheral vascular disease. Resulting perfusion maps may appear speckled and DSC signal profiles in whole brain appear shallow and/or irregular, as in Figure 7. Note the CNR is extremely low throughout the brain, and the images are uninterpretable as if the patients did not have CA administered. To enable better quality DSC acquisitions in the future, CA may be administered at a lower rate (25). Yet while the lower rate may still produce acceptable rCBV maps, the rCBF may be underestimated as the rCBF accuracy relies to a greater degree on a sufficient rate of CA injection (30, 31). Three separate cases of data collected with inconsistent CA administration resulting in weak or delayed post-bolus DSC signal profile are shown in Figure 8. While delayed (wide) post-bolus signal profiles can occur due to CA injection issues, they can also occur when the saline flush is not administered at the correct rate or immediately following CA injection. While all three cases shown have a prolonged post-bolus signal profile, only the DSC profile for the patient in the top row of Figure 8 has interpretable data due to a substantial return of the signal towards baseline. As shown, the CNR is also adequate in 84% of all voxels. Since rCBV is dependent on area under the curve and less dependent on rate of injection, it is more robust than rCBF, in such cases (30, 31). Therefore, in all cases in Figure 8 the rCBF is unreliable and uninterpretable. The rCBV maps for the other two cases are compromised due to both the lack of return towards baseline, which precludes estimation of any CA leakage effects that may have occurred. An irregular appearance of the CA post-bolus in the DSC signal profile is seen in Figure 9. This irregular pattern has been observed during manual CA injection, or when the power injector sticks or becomes jammed. Even though the DSC signal is suboptimal, adequate rCBV can be produced since the calculation is dependent on the area under the curve. However, caution should be employed when evaluating other cases for injection irregularity since it might instead be due to patient movement in which case the rCBV would likely be uninterpretable because the area under the curve would not represent true signal changes. In the example displayed, motion correction did not alter the resulting maps in a meaningful way, providing further evidence that the signal irregularity was due to an irregular rate of CA administration.

### DSC signal noise

**Figure 10:**
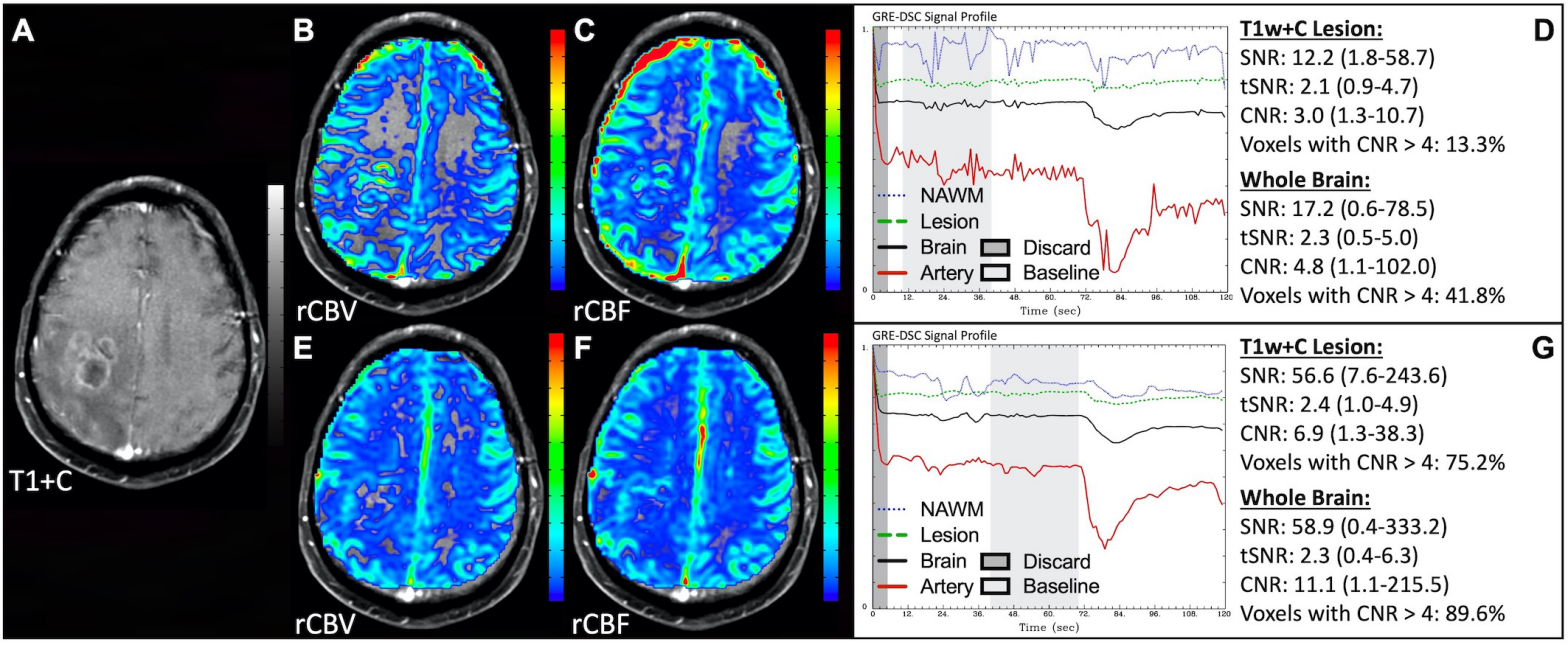
Motion and correction. Images are from a 63 y/o male with WHO 2007 grade 4 glioblastoma who was imaged shortly after having a seizure. Pictured are post-CA T1w (**A**), rCBV (**B,E**), and rCBF (**C,F**) images, and corresponding DSC signal profiles prior to (**D**) and following motion correction (**G**). As shown here, application of motion correction in the form of DSC volume registration or a smoothing filter can greatly improve CNR throughout the brain.

**Figure 11:**
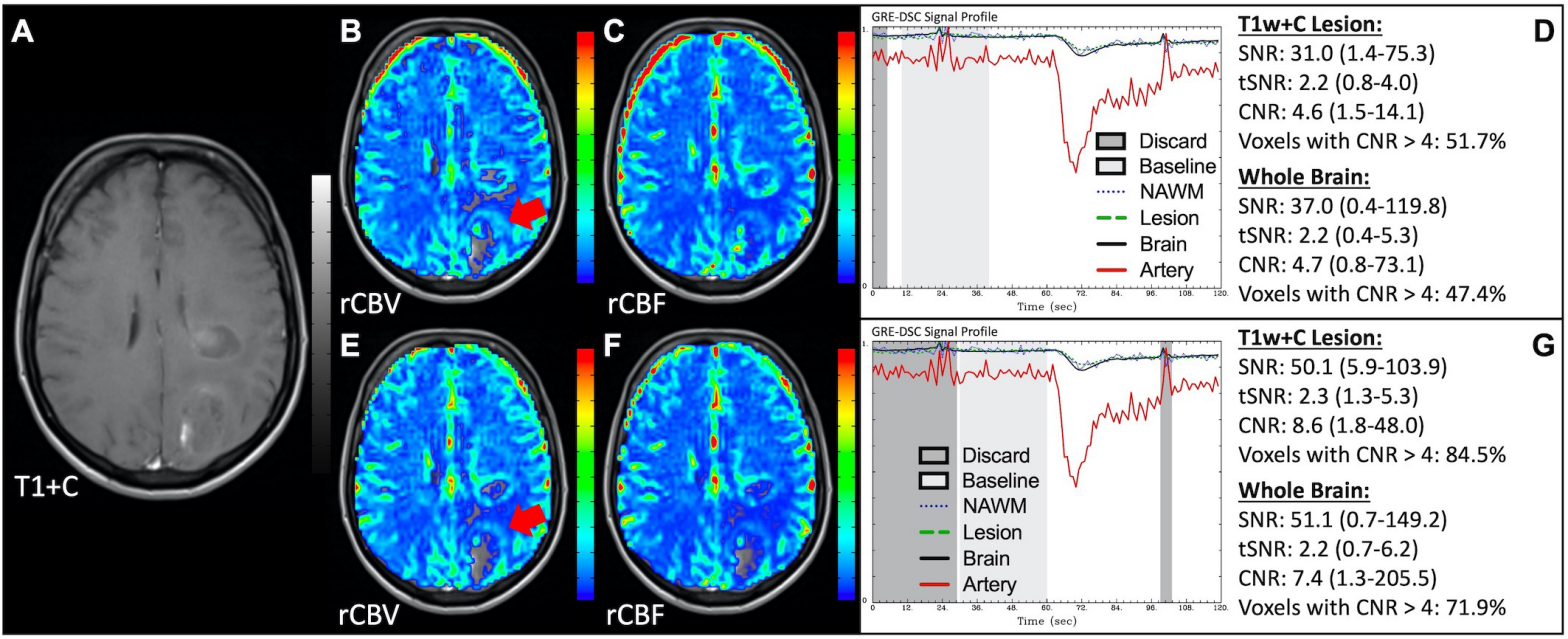
Noise spikes. Images are from a 49 y/o female with WHO 2007 grade 4 glioblastoma. Pictured are post-CA T1w (**A**), rCBV (**B,E**), and rCBF (**C,F**) images, and corresponding DSC signal profiles, respectively (**D,G**). Shifting the start and end of the region from which a mean baseline signal is computed (light gray region) and removing (**G**) DSC noise spikes (**D**) improves the perfusion maps. While the images with noise spikes are suboptimal, they are still interpretable and would have resulted in similar rCBV assessment (red arrows).

**Figure 12:**
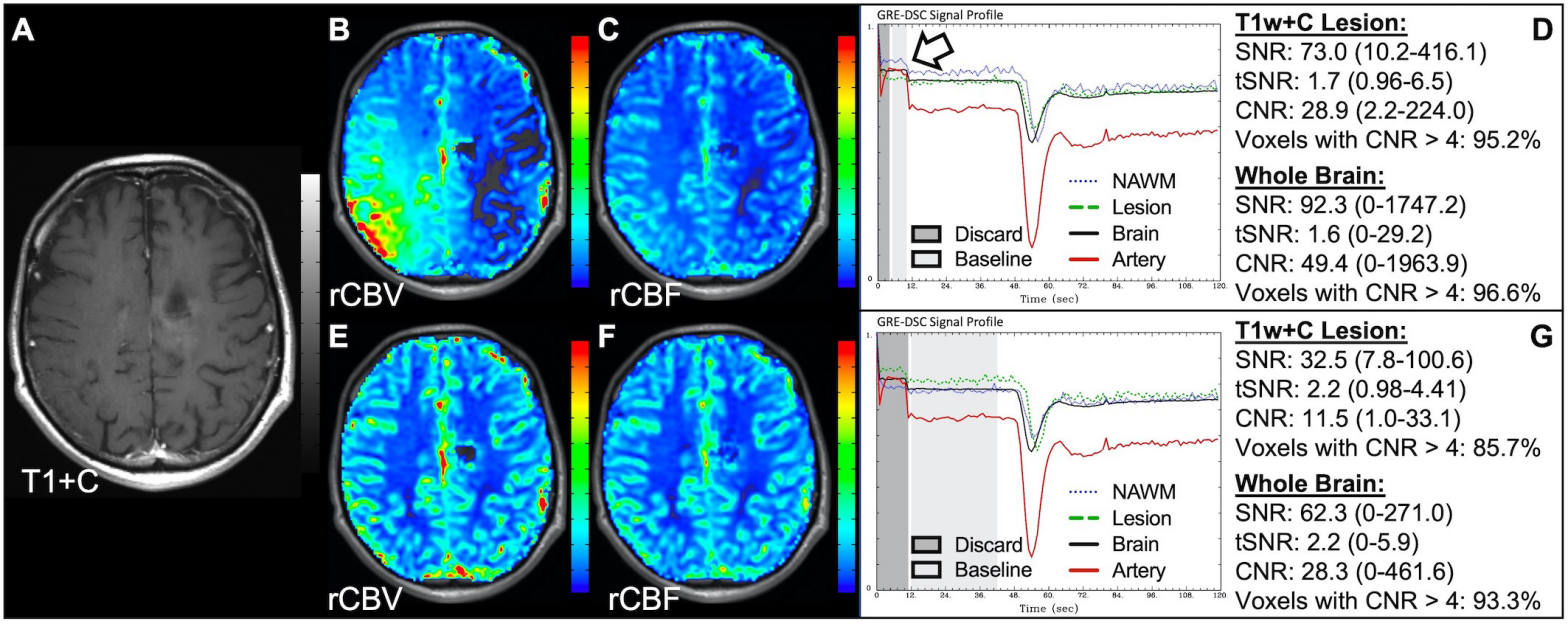
Regional DSC baseline shift. Pictured are post-CA T1w (**A**), rCBV (**B,E**), and rCBF (**C,F**) images, and corresponding DSC signal profiles (**D,G**) in a 34 y/o male patient with history of WHO 2016 grade 3 anaplastic astrocytoma. Improper inclusion (**D**) of a regional shift (arrow) in the baseline signal (light gray region) (**D**) caused markedly inaccurate calculation of rCBV (**B**), which was corrected by moving the range during which the mean baseline signal is calculated to exclude this region (**G**).

**Figure 13:**
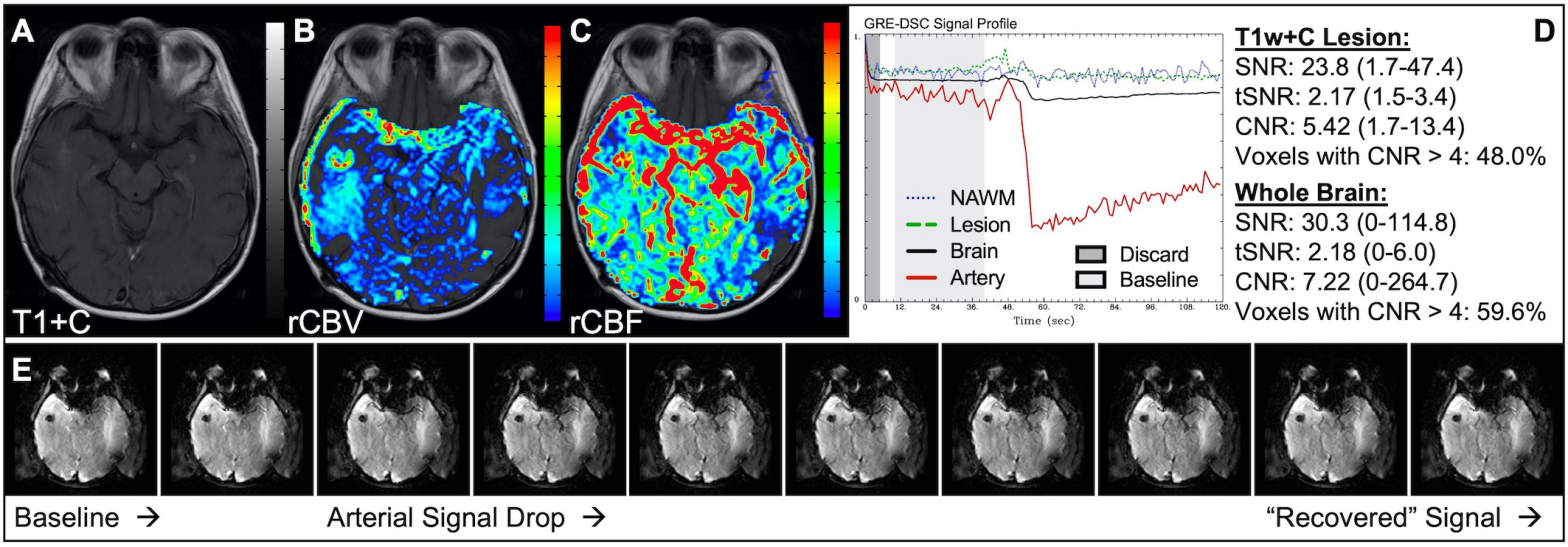
Phase-encode direction. Images are from a 46 y/o female with metastatic breast cancer. Pictured are post-CA T1w (**A**), rCBV (**B**), and rCBF (**C**) images along with the corresponding DSC signal profile (**D**) where the in-plane-phase-encode direction was incorrectly set to right-left, which resulted in severe ghosting (**E**) in the phase-encode direction making the DSC signal uninterpretable.

Data was acquired for the patient in Figure 10 shortly after experiencing a seizure, resulting in substantial motion throughout the DSC signal profile. Application of motion correction by co-registration of the DSC slices or utilizing a smoothing filter can greatly improve the CNR and resulting perfusion maps. In this case, the number of voxels in the brain with CNR > 4 was improved by 114% with motion correction with a clear improvement in the rCBV and rCBF images. Noise spikes, as seen in Figure 11 might also result in suboptimal, but still interpretable, DSC data. Depending on the dataset, large infrequent and brief noise spikes might be selectively excluded as long as the underlying DSC signal trajectory is not altered. For the case shown the noise spikes could be eliminated by adjusting the starting and ending timepoints for the images used for the calculation of the mean baseline signal (light gray region) and eliminating the images tainted by the motion spike. While the overall diagnosis would not likely change for this example, subtle differences are observed visually. Likewise, the number of voxels in the brain with CNR > 4 was improved by 52%. Adjusting the baseline is also necessary in cases such as in Figure 12, where a protracted elevation in baseline signal intensity in only one region (possibly a result of a failed coil element) resulted in perfusion maps that were initially uninterpretable. By adjusting the baseline timepoints used for the calculation of the mean baseline signal (light gray region) the effect of this regional and temporary shift could be eliminated and interpretable perfusion maps produced. In Figure 13, the poor-quality data resulted because the in-plane-phase-encode direction was incorrectly set to right-left, which resulted in severe ghosting in the phase-encode direction making the DSC signal uninterpretable.

### Susceptibility artifact

**Figure 14:**
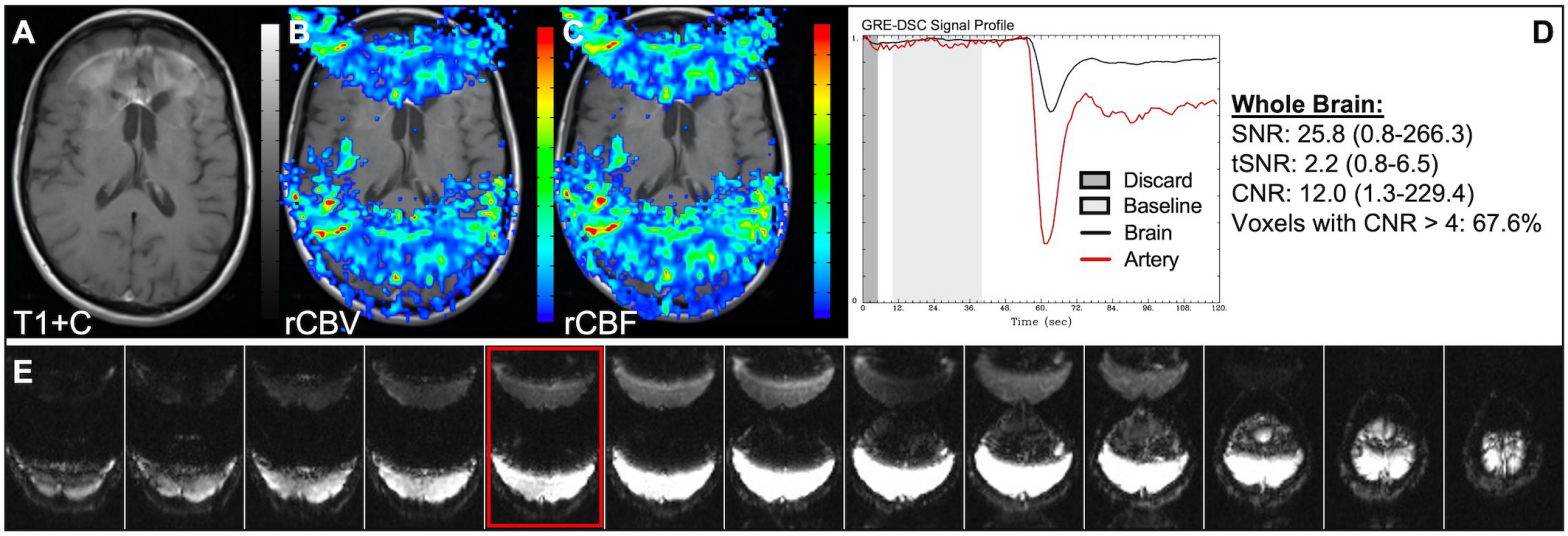
Metal artifact. Images are from a 57 y/o female with history of (pre-WHO 2007) grade 3 anaplastic oligodendroglioma. Pictured are post-CA T1w (**A**), rCBV (**B**), and rCBF (**C**) images and corresponding DSC signal profile (**D**) in a patient with artifact from metal braces. Additionally, DSC images within one baseline volume are shown (**E**), where the image outlined in red is from the same slice as (**A–C**). Depending on the region to be examined, those with metal artifacts may not be suitable for DSC acquisition. While the artifact here is more extreme, signal loss has been observed in patients with dental work, post-surgical clips, and hair extensions, among others. NOTE: rCBF is shown without normalization, as a NAWM region could not be determined.

**Figure 15:**
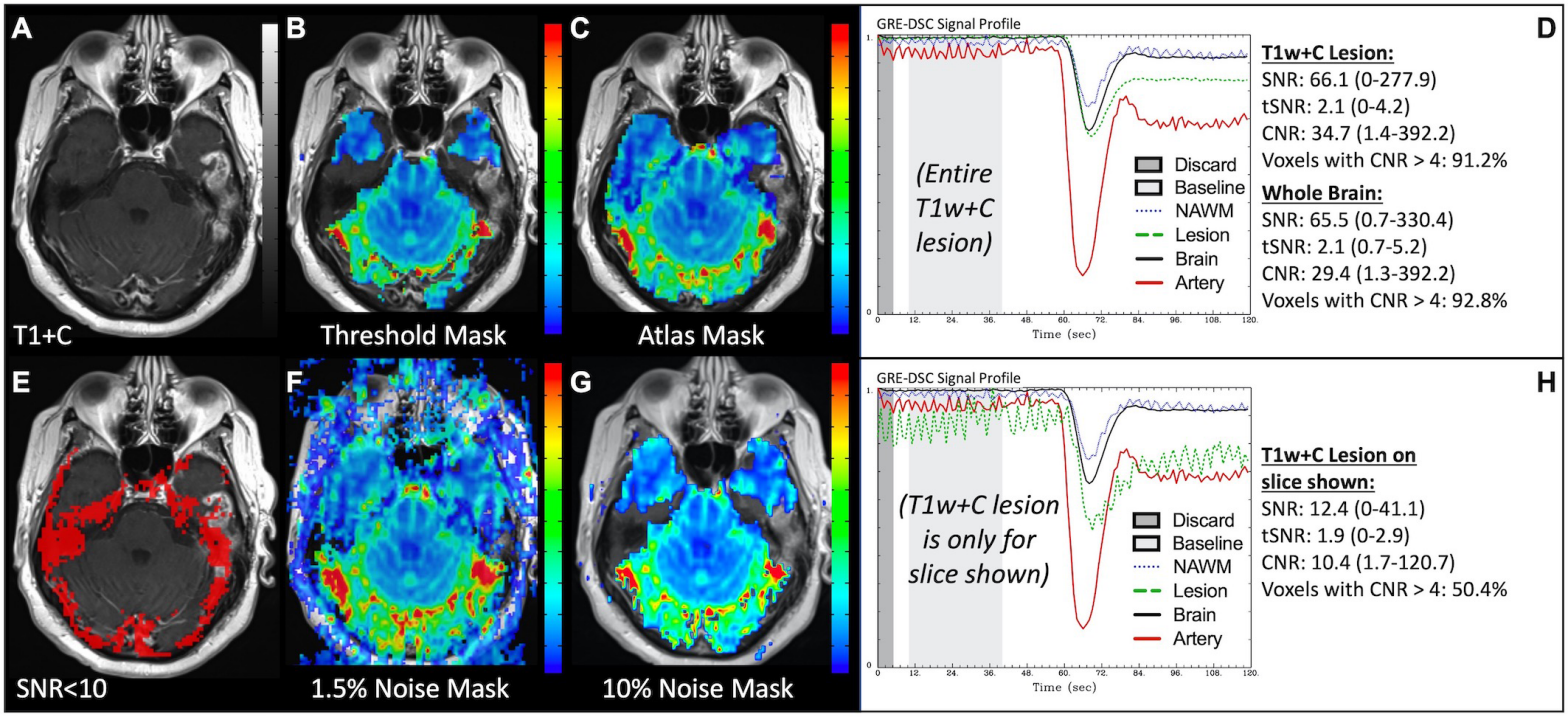
Brain masking and bone-air-tissue interface. Images are from a 46 y/o male with WHO 2016 grade 4 glioblastoma. Pictured are post-CA T1w (**A**) and rCBV (**B,C,F,G**) images with threshold-based (**B**), atlas-based (**C**), and noise-based (**F,G**) DSC brain masking, along with DSC signal profiles in the entire T1w+C lesion (**D**) and only the T1w+C lesion for the slice shown in (**A**) at the air-tissue interface (**H**). Voxels with DSC SNR <10 are shown in red in (**E**). While debated as to which masking options are best, sufficient noise-based mask options (**G**) force exclusion of invalid DSC signal, as typically occurs at the bone-air-tissue interface. DSC brain masking was manually adjusted to include all T1w enhancement, respectively, for the calculation of SNR, tSNR and CNR.

Since DSC exploits transient CA-induced changes in susceptibility between the vessel and tissue, anything which affects susceptibility may also impact these measurements. Unwanted magnetic susceptibility effects may result in T2* signal loss and distortion and can be observed in patients with, for example, implanted screws, surgical clips, blood products and shunts in addition to dental work and hair extensions. In the images in Figure 14, the susceptibility effects due to braces made the images completely uninterpretable. Similarly, Figure 15 displays how susceptibility effects can also be present towards the skull-base, near the bone-air-tissue interface. Similar effects may also be observed near resection cavities or sinuses. These distortions are reduced with SE acquisitions but at the cost of lower SNR compared to GRE acquisitions. However, it is important to ensure that when these distortions are present, voxels with faulty DSC signal are not included in the brain mask to prevent misinformed clinical interpretation. In this case, signal loss causes 50% of T1w+C lesion voxels to be uninterpretable for the slice shown. While different masking techniques offer various advantages, appropriate noise-based techniques provide a better gauge of signal quality than atlas-based masks, which though more appealing visually may over-include poor quality voxels.

A summary of all the described figures including sources of error, appearance of images, signal quality, and suggestions can be found in Table 1.

**Table 1:** Summary of proper DSC-MRI acquisition and issues described in Figs. 1–15, including sources of error, appearance of images, signal quality, and suggestions.

	Proper DSC-MRI Acquisition	Timing & Presence of CA Administration	Rate of CA Administration	DSC Signal Noise	Susceptibility Artifact
Figures	1, 2A–D, 2E–H, 3E–H, 5E–G	3A–D, 4, 5A–D, 6A–D, 6E–H	7, 8, 9	10A–D, 10E–H, 11A–D, 11E–H, 12A–D, 12E–H, 13	14, 15
Sources of Error	Not relevant	CA administered too early, too late, outside of DSC acquisition window, or with insufficient time following CA preload	CA administered manually, IV issue (leak, clamp, location, blown vein), missed or delayed saline flush, swapping of CA and saline order, poor vasculature (chronic drug abuse, Ehlers-Danlos syndrome, peripheral vasculopathy)	Intrinsic Noise, early transient noise, patient motion (long scan times, seizure, anxiety), miscellaneous (hardware issues, coil, scan settings)	Commonly observed with implanted screws, surgical clips, blood products, shunts, dental work, hair extensions, and proximity to bone-air-tissue interfaces, sinuses, or resection cavities
rCBV/rCBF Image Appearance	Good contrast between blood vessels and normal brain	Globally flat or speckled contrast	Globally flat or speckled contrast	Signal voids	Signal voids
DSC Image Appearance	Globally, blood vessels display a darkened appearance when CA passes through tissue.	Absent CA: Blood vessels do not have a darkened appearance at any point, indicating no CA was administered.	Issues may or may not be observable. Blood vessels may have absent or subdued darkening in appearance following CA administration.	Movement apparent across all or some images.	Signal voids
DSC Signal Appearance	Relatively flat baseline signal (>30 timepoints) prior to a post-bolus CA dip and signal recovery	Absent CA: Flat signal across the whole brain; Present CA: early (<30 timepoints) or delayed post-bolus signal dip	Delayed/prolonged, weak/low amplitude, and/or irregular post-bolus signal dip	Noise spikes, erratic and/or irregular signal changes	Regional signal loss, which may not be apparent globally
Average SNR (Range)	GRE: 68.1 (38.6–88.3) SE: 47.7	GRE: 47.7 (31.7–75.3) SE: 58.5	GRE: 112.3 (49.9–359.6)	GRE, Raw: 44.2 (17.2–92.3) GRE, Corrected: 57.4 (51.1–62.3	GRE: 45.7 (25.8–65.5)
Average tSNR (Range)	GRE: 2.2 (2.1–2.2) SE: 2.1	GRE: 2.3 (1.7–3.1) SE: 2.2 Early CA administration: Lower mean tSNR with a shortened baseline	GRE: 2.3 (2.1–2.6)	GRE, Raw: 2.1 (1.6–2.3) GRE, Corrected: 2.2 (2.2–2.3)	GRE: 2.2 (2.1–2.2)
Average CNR (Range)	GRE: 32.8 (14.6–57) SE: 8.8	GRE: 4.5 (2.7–8.4) SE: 3	GRE: 15.7 (3.3–73.9)	GRE, Raw: 16.5 (4.7–49.4) GRE, Corrected: 15.6 (7.4–28.3)	GRE: 20.7 (12.0–29.4)
Average Percent of Voxels with CNR > 4 (Range)	GRE: 94.0 (84.0–100.0) SE: 88.3	GRE: 32.4 (6.5–60.9) SE: 10	GRE: 62.2 (16.8–96.3)	GRE, Raw: 58.9 (41.8–86.6) GRE, Corrected: 84.9 (71.9–93.3)	GRE: 80.2 (67.6–92.8)
Suggestions	Not relevant	MR technologist should review DSC acquisition protocol prior to scanning session.	MR technologist should manually verify all IV tubing prior to scanning, review DSC protocol, and adjust CA flow rate if patient has any noted vasculopathy.	Educate patient on effects of movement prior to scan and explain scanning process to reduce patient anxiety. Apply post-processing motion correction or filters to DSC data as warranted.	Apply appropriate brain masking, especially using noise filtering, of DSC signal to ensure only quality voxels are interpreted clinically.

## Discussion

While DSC-MRI data is relatively straightforward to obtain, as described in the recently published national consensus recommendation (10), it is still possible to collect poor quality data from which suboptimal perfusion maps result. The inability to recognize a poorly acquired dataset can undermine clinical decision making. For this reason, the goal of this paper is to provide practical examples to improve our ability to recognize and correct such issues, when possible. As described, the most typical reasons for poor quality data involve CA administration that are sometimes related to human error. While many of the timing issues are easily recognizable (CA administered manually, too early, too late, outside of DSC acquisition window, or with insufficient time following CA preload), others are less common and may be encountered due to the IV (leak, clamp, location, blown vein), missed or delayed saline flush, swapping of CA and saline order, or not reducing injection rate in a patient with previously identified poor vasculature (chronic drug abuse, Ehlers-Danlos syndrome, peripheral vasculopathy). While the corruption of the DSC signal by noise or motion can be more random and thus more difficult to control, motion correction (image registration, smoothing filters), removal of data spikes, and manual selection of sufficient and correct baseline and integration limits may be performed during post-processing to improve data quality and interpretation. In addition, patient education concerning the scanning process and effects of movement during acquisition has been shown to reduce both patient anxiety and motion during the scanning session (32). Finally, noise filtering and brain masking can improve the resulting rCBV and rCBF images, increasing reliability of clinical interpretation. Additional benefits to noise or motion filtering include better automatic AIF determination, preventing an AIF from being placed in noise rather than true artery, especially when no CA has been administered.

Regardless of whether an image can be improved, it is imperative to understand and recognize issues pertaining to DSC data quality, so that uninterpretable data is not used for clinical decision making, and suboptimal data not gratuitously relied upon. In all the examples provided, the datasets can be evaluated visually for quality with a proper understanding of the DSC signal profiles.

Within the whole brain, average SNR did not effectively discern an overall good quality DSC acquisition from one with problematic issues, likely because SNR is directly related to baseline signal height. However, it may be helpful for use in brain masking and in determining individual voxels where rCBV or rCBF interpretation is unreliable due to low DSC signal. Similarly, average tSNR did not provide good discrimination between reliable and poor-quality data. Interestingly though, the only two cases where average tSNR was decreased below 2.0 occurred when the baseline signal calculation contained 10 or fewer timepoints, rather than 40, which is consistent with the recommendations of Boxerman et al. who reported that 34% more noise is present in DSC data acquired with 10 compared to 50 baseline timepoints (23).

Since CNR incorporates both baseline signal intensity and the post-bolus signal profile, CNR may provide a better indication of image quality. With the exceptions of SE data, susceptibility artifact and elevated baseline DSC signal, average DSC CNR in whole brain for uninterpretable rCBV was less than 8.5, suboptimal rCBV was between 4.7 and 74, and adequate rCBV was between 14.6 and 57. Most telling however, was the percent of voxels with CNR greater than 4. Outside of the issue described in Figure 12, related to elevated baseline inclusion, all the uninterpretable rCBV cases had less than 63% of voxels with a DSC CNR > 4, while suboptimal or adequate DSC cases had between 47%-96.3% or 84%-100% of voxels with DSC CNR > 4, respectively. Since rCBF calculation is more sensitive to post-bolus dispersion and delay, the CNR may not be a good indicator for overall data quality when those conditions occur.

Altogether, the most robust assessment of DSC signal quality is a visual assessment of the shape of the DSC signal profile, as displayed in each figure. While the presence of DSC noise or motion might make it easy to identify poor quality data, the DSC signal profile should be further scrutinized visually, and include evaluation of (1) sufficient baseline signal, (2) presence and quality of the post-bolus signal drop in arterial signal, (3) evidence of sufficient post-bolus signal drop in whole brain, and (4) a sufficient signal recovery approaching signal baseline.

Beyond the necessary evaluation of DSC signal quality, all the post-processed pMRI datasets that involved missing or suboptimal CA administration or timing issues had a visually speckled or spotty appearance on rCBV, indicating another way by which poor datasets can be identified. Otherwise, rCBF generally had a visually flatter image contrast. Occasionally these characteristics can also be seen for rCBV with very shallow post-bolus DSC signal profiles.

Alongside clinical interpretation, pMRI post-processed images should include DSC data quality assurance. While noise evaluation may be automated and might indicate something is wrong, at a minimum a snapshot of the DSC signal profile with comparison of arterial to whole brain tissue should be included with processed rCBV and rCBF images. Additionally, while only one software platform was used to generate the rCBV and rCBF images, the ability to resolve or troubleshoot issues described here is platform agnostic and dependent on the provided flexibility within a particular tool.

Although many acquisition and post-processing issues have been described, this guidance is not representative of every issue that could occur in every type of DSC sequence. However, most issues should fit within the categories identified and become more easily recognizable through an understanding of proper DSC signal characteristics.

Clearly, clinical workflows should incorporate visual inspection and assessment of DSC signal profile, image quality, and the presence of signal noise whether automated or not, with included quality assurance available to the interpreting clinician. With proper attention and informed decision, both large and small centers should be equipped to provide high quality perfusion maps using software that is clinically available.

## Data Availability

The deidentified raw data supporting the conclusions of this article will be made available by the authors upon request, without undue reservation.
